# Supplementary material for: Molecular Signature of Long Non-Coding RNA Associated with Areca Nut-Induced Head and Neck Cancer
Source: Cells. 2023 Mar 11;12(6):873. doi: 10.3390/cells12060873 (PMC10047708; doi:10.3390/cells12060873)
Supplement: Supplementary file 1 [file cells-12-00873-s001.zip › cells-2205286-supplementary.pdf]

Supplementary information:
Molecular Signature of Long Non-Coding RNA Associated with Areca Nut-Induced Head and Neck Cancer

Hung-Han Huang, Guo-Rung You, Shang-Ju Tang, Joseph T. Chang, and Ann-Joy Cheng

Cell line information

Five cell lines (OECM1, SAS, FaDu, Detroit 562 (Detroit) and CGHNC8) were used in this study. The cell line culture information was stated in the Material and Method section. Eight head and neck cancer cell pellets (CGHNC9, OC3, SCC25, SCC4, Detroit 562, NPC076, BM1, and BM2) and ten oral normal keratinocyte pellets (CGHNK2, CGHNK6, CGHNK16, K30, K43, K44, K45, K47, K48, and NOK) were used in this study. The specific information of each cell line is below.

OECM1

The OECM1 is a human oral squamous cell carcinoma cell line, which was derived from the surgical resection of a primary tumor of a male patient in Taiwan [68]. OECM-1 contains a genomic mutation in *TP53*.
Database Name: Expasy Cellosaurus
Accession Numbers: CVCL_6782

SAS

The SAS is a poorly differentiated squamous cell carcinoma cell line from a human tongue primary lesion in Japan [69, 70]. SAS contains a genomic mutation in *TP53*.
Database Name: Expasy Cellosaurus
Accession Numbers: CVCL_1675

FaDu

The FaDu is hypopharyngeal squamous cell carcinoma cell lines from a tumor of a 56-year-old male patient [71]. FaDu contains genomic mutations in *CDNK2A*, *FAT1*, and *TP53*.
Database Name: Expasy Cellosaurus
Accession Numbers: CVCL_1218

OC3

The OC3 cell line was derived from a 57-year-old man with oral squamous cell carcinoma with a long-term areca nut habit [72]. OC3 contained several mutations including the gain in chromosomes 1q, 5q, and 8q, the loss in 4q, 6p, and 8p as well as the gain of entire chromosome 20.
Database Name: Expasy Cellosaurus
Accession Numbers: CVCL_D859

SCC25/SCC4

SCC25 and SCC4 were derived from a 55-year-old and 70-year-old man with squamous cell carcinoma of the tongue, who had radiation and methotrexate treatment [73]. The genomic mutation of this cell line was *TP53*.
Database Name: Expasy Cellosaurus
Accession Numbers: CVCL_1682/ CVCL_1684

Detroit 562

The Detroit 562 is a human pharyngeal squamous cell carcinoma cell line, which is derived from the metastatic carcinomatous cells in the pleural fluid of a female, Caucasian, pharyngeal cancer patient in the USA [74]. The Detroit 562 contains genomic mutations in *TP53*.

Database Name: Expasy Cellosaurus
Accession Numbers: CVCL_1171

NPC-076
NPC-076 also called NPC-TW02 is keratinizing nasopharyngeal squamous carcinoma cell derived from a 36-year-old female [75].
Database Name: Expasy Cellosaurus
Accession Numbers: CVCL_6009

BM1/BM2
BM1 and BM2 cell lines were derived from a metastatic poorly differentiated carcinoma in the bone marrow aspirate of a 37-year-old nasopharyngeal carcinoma female [76]. These cell lines contain 21 genetic mutation including t(1;9)(p11;q11), i(3)(q10), add(4p), del(6p), del(7)(q31), i(8)(q10), t(11;22)(q11;q11), i(13)(q10), i(22)(q10), del(X)(q24), t(2;?;2)(p11.2;?;q21), t(3;?;4)(p13;?;q13), add(5)(q11), add(10)(p12), del(?)t(?;12)(?;p11), add(15)(p11), add(21)(p11), add(X)(q22), i(8q), i(13q), and i(22q).
Database Name: Expasy Cellosaurus
Accession Numbers: CVCL_6007

CGHNC8, CGHNC9, CGHNK2, CGHNK6, CGHNK16, K30, K43, K44, K45, K47, K48
These cancer or normal keratinocyte cell lines were previously established by our laboratory, from tumors or the adjacent normal mucosa tissues of patients with oral cancers [36]. Each sample used in this study was obtained before the patient's informed consent and approved by the Institutional Review Board of Chang Gung Memorial Hospital, Taiwan. The CGHNC8 or CGHNC9 cell lines were derived from patients with the habits of areca nut chewing and smoking with oral cancer [36]. The CGHNK2, CGHNK6, CGHNK16, K30, K43, K44, K45, K47, and K48 were primary culture cells from tissue biopsies of grossly normal oral mucosa with (CGHNK2, CGHNK6, and CGHNK16) or without (K30, K43, K44, K45, K47, K48) immortalization by human papillomavirus.

NOK
NOK (normal oral keratinocyte) also called NHOK (normal human oral keratinocytes) or GK (gingival keratinocyte) was derived from healthy gingival tissue during flap operation to remove the impacted third molar [77, 78]. NOK was immortalized by hTERT.

Supplementary Figure S1

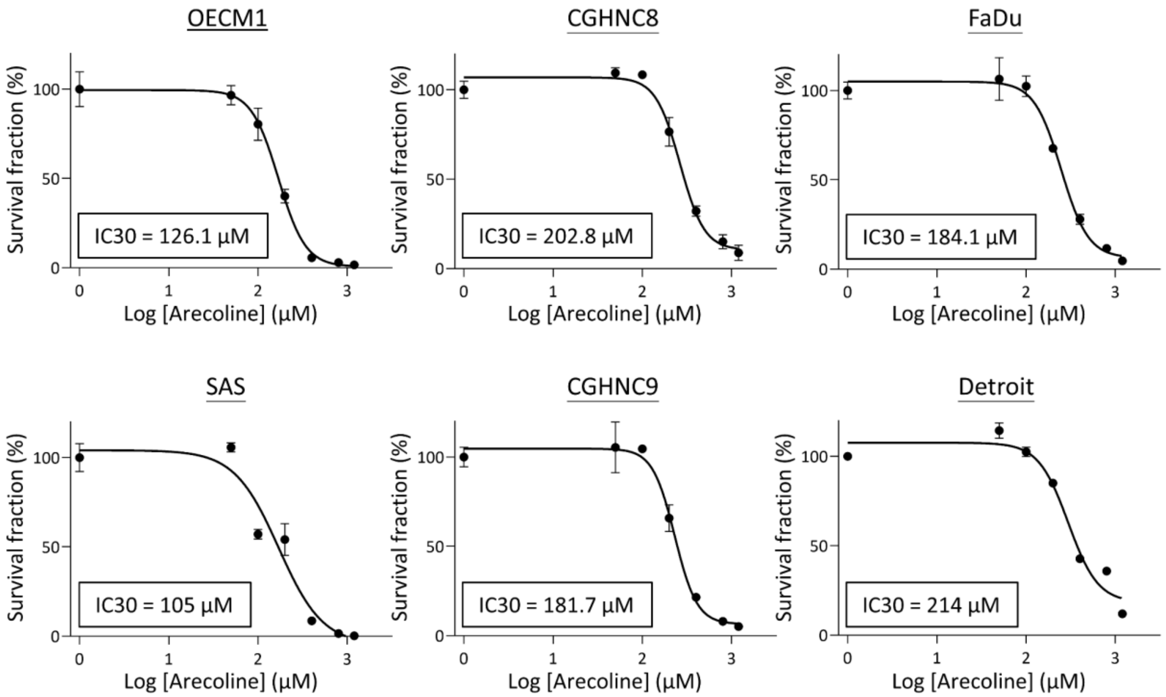

Figure S1. The IC30 dose of arecoline among six head and neck cancer cell lines. All cell lines were treated with 0, 50, 100, 200, 400, 800, 1600 μM arecoline for 24 hours. CCK8 was used to determine cell viability. IC30 in these HNC cell lines ranged approximately from 100 to 200 μM.

Supplementary Table S1. List of primers and siRNA sequence

Primer name	Sequence (Forward, 5' -> 3')	Sequence (Reverse, 5' -> 3')
LUCAT1	AGACAATGCCCAGACCTCCA	GCCCATGGTAGATGCTGAACC
HIF1A-AS2	TAAAGGACCTAAGGCTCTGGC	TGAGTGAAGCAGTTCTCAGCAT
MIR31HG	CTGGGCCCTATGGGTTTCTG	TGGTGAGCCTGCATTTAGGG
UCA1	CATCGGCTTAGCAACAGGGA	AACAAGGTGCCAGTTAGCGT
SUMO1P3	TATCGGGTTGAAGTCAAGATAACTG	ATCCCAGGCCAAAAAGTACAA
LINC00312	GATCTATGGCCCATCATTCTTT	GTCCATCATGTAGCAAGCAGT
MMP1	GCTAGCTCAGGATGACATTG	ACCTGGATCCATAGATCGTT
FLNB	GGCGCAAAAGGCAAGA	CACGTGGCTCCCATTGA
LAMA3	AGCTGCCCAAGCCAAGCAGG	TGGCGGCATCCACAGCACAG
GAPDH	GGAGCGAGATCCCTCCAAAAT	GGCTGTTGTCATACTTCTCATGG
siRNA name	Sequence (Sense, 5' -> 3')	Sequence (Antisense, 5' -> 3')
siMIR31HG	CAUCAUAGAGGAAAGACAUTT	AUGUCUUUCCUCUAUGAUGTG
